# Mineral Filler Hybridization in Recycled Polyethylene Terephthalate

**DOI:** 10.3390/polym17030259

**Published:** 2025-01-21

**Authors:** Marcel Droß, Max Ehleben, Klaus Dröder

**Affiliations:** 1 Institute of Machine Tools and Production Technology, Technische Universität Braunschweig, Langer Kamp 19b, 38106 Braunschweig, Germany; k.droeder@tu-braunschweig.de; 2 Institute for Recycling, Ostfalia University of Applied Sciences, Robert Koch Platz 8A, 38448 Wolfsburg, Germany; m.ehleben@ostfalia.de

**Keywords:** hybrid, thermoplastic resin, mechanical properties, rheological properties, particle reinforcement

## Abstract

This study focused on evaluating the mechanical, thermal, and morphological properties of recycled polyethylene terephthalate (RPET) hybrid mineral-filled composites containing fine acicular wollastonite, mica phlogopite, and talc platelets. Depending on the filler content, both single mineral-filled composites as well as hybrid mineral–filler composites were investigated. The maximum nominal filler content was set to 20% by weight with varying ratios for combinations of the wollastonite–mica and wollastonite–talc composites, respectively. Aside from the tensile, compression, and flexural properties, the heat distortion temperature and degree of crystallinity were carried out. Moreover, the dynamical response of the hybrid mineral-filled composites on different frequencies (1 Hz, 2 Hz, 5 Hz, and 10 Hz) was considered. By using scanning electron microscope photography, the fracture surface and the morphology of the composite material were observed. The results demonstrated enhanced stiffness, strengths, and thermal stability for all hybrid mineral-filled composites. In particular, the wollastonite–talc-filled RPET composites revealed a good compatibility and showed the most beneficial results.

## 1. Introduction

Polyethylene terephthalate (PET) is used worldwide and is well-known for packaging applications like beverage bottles. It is also state-of-the-art to be highly recyclable since solid-state polymerization (SSP) ensures a sufficient polymer chain length (intrinsic viscosity values > 0.8 dL/g) in order to enable the processability of recycled PET (RPET) pellets [1,2]. However, evidence shows that due to strict European Food Safety Authority (EFSA) regulations on contamination issues [3,4] and a lack of incentives to collect packaging waste (like the German deposit return scheme), the European recycling rate is only around 50%, and of that, only 17% of RPET is reused for new bottles [5]. The RPET not suitable for bottle replication is repurposed for other industrial applications such as clamshell packaging, fabric in clothing, or films used for adhesive tapes. Given the short lifespan of these products and the fact that most of them are incinerated for high-energy recovery at the end of their life, it results in a substantial waste of resources. As a consequence, the challenge is to identify alternative applications for RPET and to qualify the material for these. One promising approach appears to be the automotive sector, which will require an average share of 25% of post-consumer polymers in new vehicles from 2030 onward due to the new end-of-life vehicle regulation (amendment of Directive 2000/53/EC) [6]. In fact, recent studies show that RPET and its based fiber-reinforced polymers are already being used in modern vehicle interiors and exteriors [7]. The technical application of PET in terms of mechanical performance and thermal stability is similar to that of polyamides, but since widely used aliphatic polyamides, such as PA6 and PA6.6, are likely to be landfilled or energetically recycled, PET and its recycled material offer a more sustainable solution for future applications [8]. To address the requirements of automotive applications and to overcome the degradation of PET during recycling, synthetic glass fiber reinforcements are commonly added to the matrix, providing processability and operational safety [9,10,11,12]. However, short carbon fiber-reinforced RPET composites made from post-consumer bottle waste have also been investigated, showing significant improvements in mechanical properties with a peak at 20% in weight [13].

The use of these synthetic fibers in composites offers a wide range in benefits. However, for economic and ecologic reasons, studies are directed toward the advantages of less expensive mineral-filled or environmentally friendly mineral fiber-reinforced composites. A RPET-polypropylene blend filled with ultrafine wollastonite was prepared by Chaiwutthinan et al., resulting in increased tensile strengths, tensile modulus, and heat distortion temperature (HDT) compared with the neat RPET [14]. Incorporating talc into RPET has also been shown to improve the tensile modulus, HDT, and dynamic mechanical properties [15]. When electrical properties are to be improved, in addition to mechanical properties, mica has shown effective results [16]. A comprehensive investigation was conducted by Kráčalík et al. by preparing RPET/organoclay nanocomposites and testing the rheological and mechanical properties [17]. The results mainly showed an improvement in stiffness to the detriment of extensibility. An exception was made for silanized organoclay-filled nanocomposites (modified by (3-glycidoxypropyl) trimethoxy silane), which offered a combination of high tensile modulus and large elongation at break due to enhanced interfacial adhesion. This can be explained by the improved polar behavior, matching the natural polarity of PET. Alongside the use of mineral particle fillers, the effect of basalt fibers in RPET compared with glass fibers was explored and showed comparable improvements in tensile modulus and strength without a significant reduction in other properties [18]. Despite the fact that glass fibers are still more affordable and accessible than basalt fibers, it is predicted that basalt fibers will be one of the most prominent materials to displace traditional glass and carbon fibers in renewable energy applications [19]. Further motivated by ecological considerations, more and more studies are being conducted on the use of natural fibers in RPET. However, the poor interfacial adhesion between the hydrophilic fibers and hydrophobic polymer is a limiting factor. Furthermore, the fibers decompose during processing due to the high melting temperature of RPET. Therefore, the focus of most studies has been on the use of coupling agents and compatibilizers [20].

Since all reinforcements, whether synthetic, mineral, or natural, have their pros and cons, choosing the right combination makes it possible to highlight the advantages of each. So-called hybrid composites use more than one single type of reinforcement. The concept of combining fibrous reinforcements featuring a high aspect ratio with particulate minerals as reinforcing fillers to achieve a sufficient improvement in the mechanical properties by avoiding excessive anisotropic phenomena has been known for quite some time [21]. Since then, various research activities have been carried out in this field, and fillers with different aspect ratios and a wide range of physical properties have been combined. For example, polypropylene was filled with a glass fiber/talc combination, achieving mechanical properties that exceeded expectations based on the rule of mixtures [22]. Other studies combined the use of glass fibers with wollastonite [23] as well as talc and wollastonite [24]. It is also worth noting that research has been conducted into the combination of different types of fibers. In particular, investigations have been carried out into the combination of glass and various types of natural fibers [25,26,27,28]. Moreover, studies have already been performed on the use of synthetic fibers in combination with basalt fibers [29,30].

In this study, a hybrid usage of wollastonite (W), mica (M), and talc (T) in RPET was investigated in terms of the mechanical, thermal, and morphological properties in order to render RPET economically and ecologically more relevant for the automotive industry. For this, 20% by weight mineral-filled RPET composites of each filler were prepared using the melt-compounding method. The impact of each mineral, in addition to hybrid approaches, was investigated with regard to the mechanical and thermal characteristics. This entailed the execution of a series of tests, encompassing tensile, compression, flexural, and dynamic mechanical analysis, along with differential scanning calorimetry and HDT tests.

## 2. Methodology

### 2.1. Materials

Recycled polyethylene terephthalate (MOPET-GREEN SSP 0.85+) was used as a polymer matrix for composite testing. The material originated from MORSSINKHOF-RYMOPLAST (Lichtenvoorde, The Netherlands) and was manufactured from natural and green colored post-consumer and post-industrial PET-A scraps by extrusion with high vacuum degassing, melt filtration, and SSP (intrinsic viscosity above 0.85 dL/g). Commercial long-needled wollastonite (TREMIN^®^ 939-300), platelet-shaped mica phlogopite (TREFIL^®^ 1232-400), and fine, flaky talc (EX-GT 10) was provided by HPF THE MINERAL ENGINEERS (Quarzwerke GmbH, Frechen, Germany). The average aspect ratio of the minerals varied, being 6:1 for wollastonite and 1:30 for both mica and talc. In contrast, the densities of the minerals were quite similar, with a range of 2.85 g/cm^3^ for wollastonite, 2.8 g/cm^3^ for mica, and 2.7 g/cm^3^ for talc. The average particle size was 30 µm for wollastonite, 44 µm for mica, and 2.7 µm for talc.

### 2.2. Specimen Preparation

Before processing, the RPET granules were dried to 0.05% relative humidity at 170 °C in an oven for 7 h. Afterward, the compounds were produced using a KRAUSSMAFFEI BERSTORFF twin-screw extruder (KraussMaffei Extrusion GmbH, Laatzen, Germany) and a Scholz gravimetric feeder (Scholz Dosiertechnik GmbH, Großostheim, Germany) to ensure precise mixing of the composite materials. The compounding process was performed at 255–280 °C from the feed zone to the die, with a screw speed of about 124 rpm. The RPET composites were injection-molded to specimens according to ISO 527-2 [31] using an ARBURG 420 C (ARBURG GmbH + Co KG, Loßburg, Germany) 1000-250 at 260–285 °C in accordance with ISO 294-2 [32] and ISO 20028-2 [33]. The mold temperature was set to 120 °C. The composition of the composite materials and their designations are given in Table 1.

### 2.3. Method of Testing

#### 2.3.1. Density

The density of the manufactured composites was determined in accordance with ISO 1183-1 [34] using the immersion method at room temperature (method A). The liquid utilized for the measurement was isopropanol. The weights were taken on an analytical balance (d = 0.1 mg) from the company KERN (Kern GmbH, Großmaischeid, Germany).

#### 2.3.2. Mechanical Tests

Mechanical test specimens were conditioned at 23 °C/50% relative humidity for a minimum of 48 h in accordance with ISO 291 [35] for test room conditions. Tensile tests were performed following ISO 527-2 using a ZWICK/ROELL (ZwickRoell GmbH & Co. KG, Ulm, Germany) universal testing machine, model Z100. The tests were carried out with an initial force of 20 N. The tensile modulus was determined at a test speed of 1 mm/min via the secant slope between 0.05% and 0.25% elongation. The tensile strength was then measured at a speed of 50 mm/min. Compression tests were performed according to ISO 604 [36]. Here, the compression modulus was measured using test sample type A with an initial force of 25 N at a speed of 1 mm/min. The compression strength was determined with test sample type B at 5 mm/min and an initial force of 20 N. Flexural properties were measured according to ISO 178 [37] using a ZWICK/ROELL universal testing machine, model Z2.5, at a test speed of 2 mm/min and a support span length of 64 mm.

#### 2.3.3. Dynamic Mechanical Analysis (DMA)

The storage modulus, loss modulus as well as the damping factor (tan δ) of the composites were measured as a function of temperature in a range of 24 °C to 160 °C, using a NETZSCH DMA testing machine, model 242 E Artemis (NETZSCH-Gerätebau GmbH, Selb, Germany), equipped with a single cantilever bending fixture and free pushrod at frequencies of 1, 2, 5, and 10 Hz. With regard to [38] the T_g_ at the peak of tan δ was investigated at different frequencies to obtain more information about the interfacial interactions between the polymer matrix and the filler particles.

#### 2.3.4. Thermal Tests

The HDT of the RPET and RPET composites was carried out following ISO 75-2 [39] using a COESFELD VICAT HDT (Coesfeld GmbH & Co. KG, Dortmung, Germany) testing machine at a heating rate of 2 °C/min at 1.8 MPa. The test specimen dimensions were 80 mm × 10 mm × 4 mm. To characterize the melting and crystallization behavior of the composites, the samples were tested in a NETZSCH PROTEUS 80 (NETZSCH-Gerätebau GmbH, Selb, Germany) according to ISO 11357-1 [40] in a temperature range of 90 °C to 270 °C following a heat–cool–heat cycle at a heating and cooling rate of 10 °C/min. The degree of crystallinity was calculated by the equation:

(1) $$X_{C}=\frac{100}{{wt\%}_{RPET}}\;\frac{∆H_{m}}{∆H_{0}}$$

where *X_C_* is the crystalline fraction of the matrix, *wt*%*_RPET_* is the mass concentration of RPET excluding the filler content, ∆*H_m_* is the experimental melting enthalpy, and ∆*H*_0_ is the theoretical melting enthalpy of 100% crystalline RPET, stated to be 140 J/g according to [41].

#### 2.3.5. Morphology of Composites

The composite structures were analyzed by scanning electron microscopy (SEM) using an FEI QUANTA FEG 650 scanning electron microscope (Thermo Fisher Scientific GmbH, Dreieich, Germany). The samples were fractured, and SEM microphotographs of the surface areas were obtained. The SEM pictures were secondary electron images taken at an acceleration voltage of 10 kV and 15 kV.

## 3. Results

### 3.1. Thermal Properties

Thermal characterization of the neat RPET and single mineral-reinforced RPET composites is shown in Table 2. For all composites, the heterogenous nucleating effect of the particles led to an increased degree of crystallinity. The melting temperature (T_m_) of the composites was within the same range between 250 °C and 252 °C and were increased in comparison to the T_m_ of neat RPET, which was at 247.9 °C. For neat RPET at 130.4 °C, a cold crystallization peak could be observed from the differential scanning calorimetry (DSC); for the RPET composites, no other peaks were detectable. This was due to earlier and better crystallization of the RPET affected by more spherulite formation around the mineral particles.

The HDT was improved for all mineral-filled RPET composites. In particular, the addition of talc increased the thermal flexural stability by 36.8% up to 93.6 °C. It is interesting to note that mica did not affect the HDT in the same way, despite the fact that it exhibits a similar aspect ratio and plate-shaped geometry. It is assumed that mica platelets slide along the matrix due to their orientation, size, and poor interfacial adhesion, resulting in a reduced mobility restriction of the molecular chains along the planes compared to talc [16]. In addition, it is well-known that HDT is related to both the stiffness and viscosity of a composite, and that small particle size fillers tend to increase the viscosity more significantly [42].

### 3.2. Mechanical Properties

**Figure 1:**
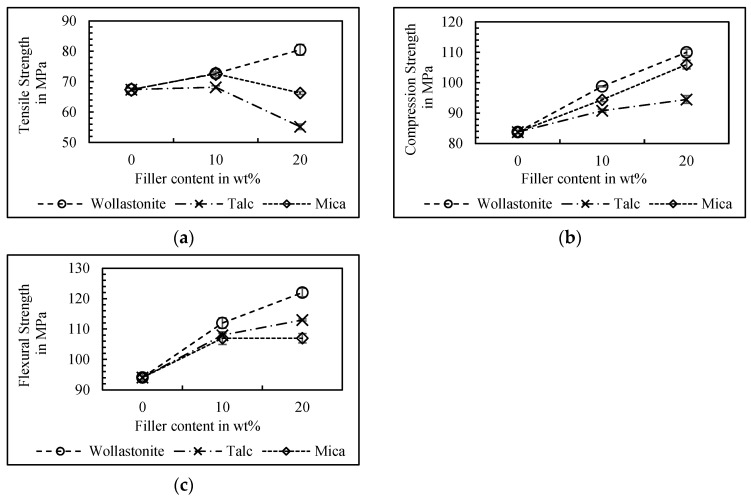
Tensile strength (**a**), compression strength (**b**), and flexural strength (**c**) of the unfilled RPET and single mineral-reinforced RPET composites.

The mechanical properties of the tested neat RPET and single mineral-reinforced RPET composites are shown in Table 3 and Figure 1. For all mineral-reinforced composites, an enhanced stiffness with a moderate density increase could be achieved. The wollastonite-reinforced composites enhanced both the strength and stiffness. The talc-filled composites showed almost no change in the tensile strength for RPET/T10 and led to a decreased tensile strength of 18% at T20. Thereby, the test sample fracture surfaces showed air bubbles, which can mainly be explained by a lack of adequate interfacial adhesion. Due to having the same aspect ratio with different particle sizes, the mica-filled composites behaved the same as the talc-filled composites. However, aside from a small peak at M10, almost no change in tensile strength was achieved. The flexural strength and flexural modulus of RPET increased with the addition of all different minerals.

While the wollastonite-filled composites enhanced the flexural strength with an increased filler content up to 22.8% for W20, T20 led to an increase of 16.7%. For the mica-filled RPET, the flexural strength was increased to 12% at M10, but could not be further improved with increasing filler content. This was due to the trend of decreased elongation at break and voids, which could be observed at the tested mica-filled sample fracture surfaces. In [16], the observation of voids, or air bubbles, a poor filler matrix adhesion, and brittle fracture behavior were already seen for the mica-filled polyvinyl chloride (PVC). It was shown in the SEM images that the mica particles were randomly oriented and underwent delamination due to tensile stresses, which took effect vertically on their flat surfaces. Nevertheless, the effect of the talc- and mica-filled RPET increasing the flexural strength at decreased tensile strength can basically be reasoned by improved compression properties due to the load-appropriate geometry and reduced mobility of the molecular chains.

The results obtained by mechanical testing of the hybrid mineral-filled RPET composites are shown in Table 4. In comparison to single mineral-filling, similar enhancements could be determined for the moduli as well as strengths. Nevertheless, half the share of wollastonite combined with talc or mica led to almost the same modulus and strength as with the single reinforcement. Moreover, the W/T filler combinations affected the stiffness and strength more than the W/M filler combinations. This phenomenon can be attributed to the combination of small particles with disparate aspect ratios (acicular and lamellar), which gives rise to a reduction in mechanical anisotropy and an elevated packing degree of the hybrid fillers.

With regard to the W5/M10 and W10/M5 specimens, it is evident that filler hybridization allowed for the adaptation of the composite to align with specific requirements while maintaining the composite’s overall density. By incorporating more wollastonite particles, better strength values could be achieved. This finding is probably due to the oriented particles parallel to the tensile loads, with delamination occurring for the mica-filled composites and favoring the load absorption of the fine acicular wollastonite particles. For the composite containing more mica-platelet particles, higher stiffness values were obtained due to the high stiffness of the filler, which is expected to limit the mobility of the RPET adjacent to the filler particle surface.

**Figure 2:**
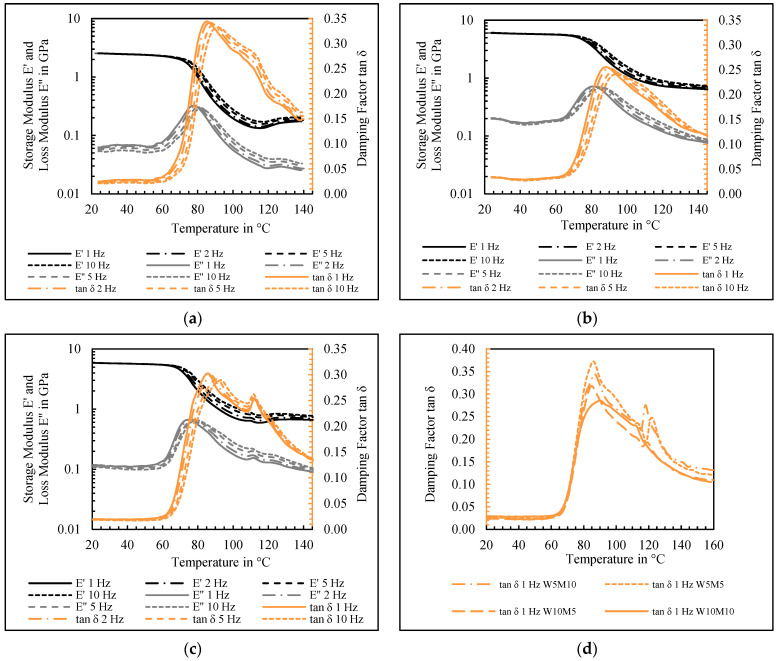
Effect of filler hybridization and frequency on the dynamic mechanical properties of (**a**) neat RPET, (**b**) the RPET/W10/T10 composite, (**c**) RPET/W10/M10 composite, and (**d**) various hybrid-filled composites of RPET/W/M.

In order to investigate the interfacial interactions of fillers and polymer, a series of DMA experiments were carried out at varying frequencies. The findings for neat RPET, W10/M10, and W10/T10, in addition to the damping factor for various RPET/W/M composites, are illustrated in Figure 2.

For the neat RPET (Figure 2a), a typical lightly cross-linked behavior was detectable, which was due to the slow nucleation of PET. At 130 °C, the cold crystallization triggered additional cross-linking and led to a slight increase in the storage modulus. It can be seen that increased frequency led to responses for the moduli and thus for tan δ, respectively. The elevated oscillation frequencies resulted in augmented T_g_ and a more elastic (solid-like) behavior in the transition region due to the diminished polymer relaxation rates. In comparison, the RPET/W10/T10 composite (Figure 2b) exhibited markedly enhanced cross-linking, in addition to a heightened storage modulus. Furthermore, the decreased T_g_ indicates a favorable interfacial interaction between the hybrid mineral fillers and the polymer matrix. For the RPET/W10/M10 composite (Figure 2c), similar improvements in storage modulus and cross-linking could be achieved. However, an effect of a second peak for T_g_ at 112 °C occurred. With respect to the three-fraction microstructure model of RPET described in [43,44], it is suggested that this effect is caused by two different T_g_ dynamics. On the one hand, a polymer–polymer interaction occurs, and on the other hand, a polymer–particle interaction takes place among the mobile and rigid amorphous phases with mineral filler particles. In this context, the rigid amorphous fractions (RAFs) are formed by macromolecules in the interface between the crystalline and mobile amorphous fractions (MAFs). Thus, the high T_g_ dynamic can be explained by the hindered mobility of the mobile amorphous phase between the lamellar regions as a result of the rigid amorphous phase. The second T_g_ dynamic is related to the bulk-like MAF. In [45], the determination of RAFs, MAFs, and the crystalline fraction by means of DSC analysis was presented using the heat capacity information provided. Against the background of the three-fraction model, Figure 2d shows the effect occurring for different RPET/W/M composites in varying degrees of intensity. Despite the fact that W5/M5 and W5/M10 caused a higher secondary T_g_ peak when compared, no clear correlation with one or the other mineral filler could be identified.

### 3.3. Morphology

**Figure 3:**
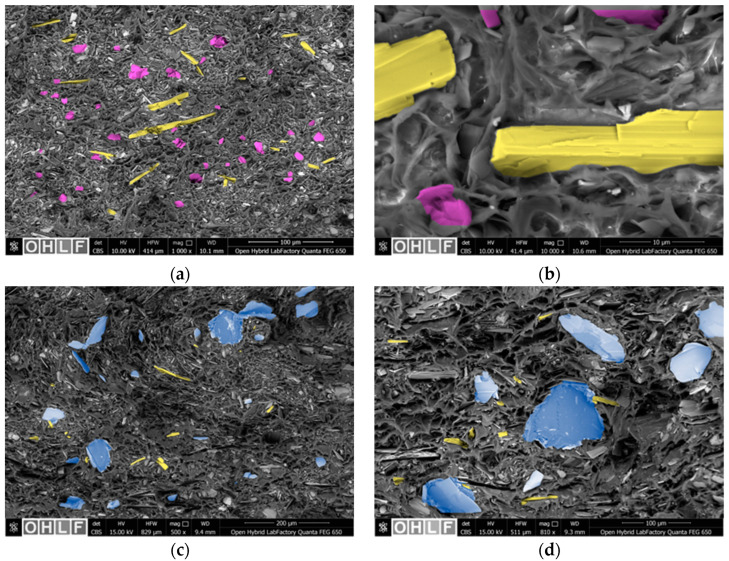
SEM micrographs of RPET/W10/T5 at 10 kV acceleration voltage showing (**a**) the filler dispersion and (**b**) the fibrillation fracture surface as well as of RPET/W10/M10 at 15 kV acceleration voltage showing (**c**) the filler dispersion and (**d**) the fibrillation fracture surface. To assist in the visual identification of the fillers, they have been partially color-coded: yellow for wollastonite, purple for talc, and blue for mica.

In Figure 3, the SEM images of the RPET/W10/T5 and RPET/W10/M10 composites at different magnifications and acceleration voltages are shown. In Figure 3a, good wollastonite and talc particle distribution could be observed while both mineral particles were well-embedded within the RPET matrix. A fibrillation fracture surface can be seen in Figure 3b, which was already described for wollastonite-filled polypropylene composites in [46]. This is due to the low strain and transition regimes around the mineral particles with wedge and ridge modes of deformation occurring. Under load, stress concentrations occur around the particles, causing the ridges to tear due to the existing deformations, stretching them and subsequently forming a multitude of fibrils with voids between them as the mineral particles detach. In this context, it is worth mentioning that the fibrils take on a cellular mesh structure due to the platelet-shaped talc particles and their aspect ratio. The same behavior and fracture surface can be seen in Figure 3c for the RPET/W10/M10 composites. However, Figure 3d depicts regions in which a similar particle orientation of the different platelets leads to direct particle–particle interaction and agglomeration of the stacked particles. Due to this, reduced particle–polymer interaction can take place, resulting in delamination and poor adhesion of the filler matrix under load.

## 4. Conclusions

In this study, single and hybrid mineral-filled RPET composites incorporating fine wollastonite, mica phlogopite, and talc were investigated. Based on the mechanical and thermal characterization as well as morphological observations, a comparison of their influences on stiffness, strength, HDT, and thermal and crystallization behavior was presented. The results of neat RPET were compared with those of the single mineral-filled RPET and hybrid mineral-filled RPET. For the wollastonite-filled RPET composites, an increased stiffness, strength, and HDT was attained while the mica-filled RPET composites and talc-filled RPET composites improved their flexural properties only through enhanced compression modulus and strength. For the mineral platelets, a typical decrease in tensile strength occurred at a weight fraction of 20%. By comparing the hybrid mineral-filled RPET composites to each other, the wollastonite–talc combinations achieved the highest flexural strength. While comparing them with single mineral-filled composites, it was found that half the share of wollastonite combined with talc or mica led to almost the same modulus and strength as with a single reinforcement. In addition, the favored particle–particle interaction of the wollastonite–talc combination resulted in improved mechanical stiffness due to increased packing efficiency. The DMA analysis and SEM images revealed good particle–polymer interaction for the RPET/W/T combinations as well as evidence of stress concentrations around the mineral particles by causing a fibrillation fracture surface. With respect to the results achieved for the PA6/W20/T20 composites in [24], the hybrid mineral-filled RPET composites with a nominal 20 wt% fillers incorporated showed similar results for flexural properties with less filler content. In order to present a sustainable solution for government requirements of the automotive industry through the enlarged application of post-consumer polymers in future vehicles, hybrid mineral-filled RPET composites can replace engineering polymers such as virgin polyamides and add significant advantages like reduced carbon dioxide equivalents, good recyclability, and reduced filler costs with simultaneously improved mechanical and thermal properties.

## Figures and Tables

**Table 1:** Nominal weight and calculated real volume contents of filler in the RPET composites.

Nominal Filler Content (wt%)	Composite Description (vol%)			
	Wollastonite (d_50_ = 30 µm)	Mica (d_50_ = 44 µm)	Talc (d_50_ = 2.7 µm)	Hybrids
10	RPET/W10 (4.90)	RPET/M10 (5.12)	RPET/T10 (5.31)	RPET/W5/T5 (4.78) RPET/W5/M5 (4.75)
15	-	-	-	RPET/W5/M10 (7.59) RPET/W10/T5 (7.61) RPET/W10/M5 (7.58)
20	RPET/W20 (10.61)	RPET/M20 (10.90)	RPET/T20 (10.77)	RPET/W10/M10 (10.59) RPET/W10/T10 (10.77)

**Table 2:** Degree of crystallinity and heat distortion temperature of the neat RPET and singe mineral-reinforced RPET composites.

Description	Crystallinity (%)	T_m_ (°C)	HDT (°C)
RPET	20.68	247.9	68.4
RPET/W10	28.03	251.4	79.2
RPET/M10	29.36	251.9	76.6
RPET/T10	29.48	249.9	83.9
RPET/W20	29.19	251.5	84.6
RPET/M20	30.84	251.4	84.9
RPET/T20	27.80	250.5	93.6

**Table 3:** Density, tensile, compression, and flexural modulus of the unfilled RPET and single mineral-reinforced RPET composites.

Description	Density (g/cm^3^)	Tensile Modulus (GPa)	Compression Modulus (GPa)	Flexural Modulus (GPa)
RPET	1.357 ± 0.000	3.250 ± 0.129	3.010 ± 0.134	2.590 ± 0.030
RPET/W10	1.444 ± 0.001	4.330 ± 0.061	4.560 ± 0.149	3.880 ± 0.106
RPET/M10	1.440 ± 0.009	4.450 ± 0.111	4.810 ± 0.131	3.990 ± 0.116
RPET/T10	1.445 ± 0.003	4.210 ± 0.066	4.400 ± 0.206	3.990 ± 0.071
RPET/W20	1.527 ± 0.003	5.920 ± 0.033	6.470 ± 0.279	5.260 ± 0.106
RPET/M20	1.526 ± 0.002	6.210 ± 0.288	6.730 ± 0.337	5.930 ± 0.113
RPET/T20	1.523 ± 0.001	5.820 ± 0.157	6.050 ± 0.197	5.540 ± 0.042

**Table 4:** Nominal weight and calculated real volume contents of filler in the RPET composites.

Nominal (wt%) Filler Content	Description	Density (g/cm^3^)	Tensile Modulus (GPa)	Tensile Strength (MPa)	Compression Modulus (GPa)	Compression Strength (MPa)	Flexural Modulus (GPa)	Flexural Strength (MPa)
10	RPET/W5/M5 RPET/W5/T5	1.438 ± 0.001 1.444 ± 0.002	3.910 ± 0.217 3.810 ± 0.053	74.5 ± 0.8 75.0 ± 0.4	4.390 ± 0.115 4.240 ± 0.343	96.1 ± 0.5 96.6 ± 0.8	3.690 ± 0.117 3.720 ± 0.163	109 ± 1.9 116 ± 0.8
15	RPET/W5/M10 RPET/W10/T5 RPET/W10/M5	1.478 ± 0.001 1.509 ± 0.002 1.481 ± 0.004	4.850 ± 0.165 5.190 ± 0.145 4.670 ± 0.242	75.5 ± 0.2 78.8 ± 0.3 77.5 ± 0.7	5.010 ± 0.376 5.430 ± 0.152 4.870 ± 0.186	101 ± 1.0 105 ± 0.5 101 ± 1.2	4.600 ± 0.171 4.780 ± 0.162 4.210 ± 0.128	110 ± 2.1 123 ± 0.6 116 ± 0.3
20	RPET/W10/M10 RPET/W10/T10	1.509 ± 0.001 1.543 ± 0.002	5.640 ± 0.166 5.950 ± 0.180	79.3 ± 0.9 79.0 ± 0.9	5.590 ± 0.351 6.160 ± 0.175	106 ± 1.0 107 ± 1.7	5.200 ± 0.204 5.710 ± 0.236	117 ± 0.9 122 ± 1.44

## Data Availability

The original contributions presented in this study are included in the article. Further inquiries can be directed to the corresponding author.

## Abbreviations

The following abbreviations are used in this manuscript:

DMA	Dynamic mechanical analysis
DSC	Differential scanning calorimetry
HDT	Heat distortion temperature
MAF	Mobile amorphous fractions
PA	Polyamide
PET	Polyethylene terephthalate
RAF	Rigid amorphous fractions
RPET	Recycled polyethylene terephthalate
SEM	Scanning electron microscope
SSP	Solid-state polymerization
